# Prediction of the Global Potential Distribution Area of *Phytopythium litorale* Based on the Maxent Model

**DOI:** 10.3390/biology15120916

**Published:** 2026-06-11

**Authors:** Junli Feng, Xiaorui Zhang, Yifan Ding, Bingyan Zheng, Jiahui Zang, Chun Yang, Haiwen Wang, Yuzhe Kong, Tingting Dai

**Affiliations:** Co-Innovation Center for the Sustainable Forestry in Southern China, Nanjing Forestry University, Nanjing 210037, China

**Keywords:** *P.* *litorale*, MaxEnt, suitable habitat, global distribution

## Abstract

This study aims to predict the global distribution and future potential distribution trends of *Phytopythium litorale* to support its prevention and control. The current suitable habitat area is estimated at 3702.85 × 10^4^ km^2^, mainly in North America, Europe, Asia, and Oceania. Using 150 global occurrence records and 36 environmental variables, we applied the MaxEnt model (FC = LQHPT, RM = 4) to identify dominant environmental factors and project suitable habitats and centroid shifts under three future climate scenarios. The model performed well (AUC > 0.95). The main influencing factors were Mean Temperature of the Coldest Quarter, Isothermality, and Precipitation of the Coldest Quarter. Under future scenarios, the total suitable area is projected to increase, while some currently suitable regions will shrink and shift toward higher latitudes. The habitat centroid is predicted to migrate northeastward from Tamanrasset Province, Algeria, with a maximum displacement of 960 km (SSP3-7.0, 2081–2100). By predicting these distributional shifts, this study enhances our understanding of *P. litorale* biogeography and provides a scientific basis for managing this pathogen under climate change.

## Simple Summary

Climate change is changing the environment where many harmful microorganisms live. This study focuses on a pathogen called *Phytopythium litorale*, which may threaten plant health and natural ecosystems. By analyzing 150 global occurrence records and 36 environmental factors, we used a computer model to predict its suitable habitats. The results show that the current suitable area is about 37.03 million square kilometers, mainly in North America, Europe, Asia, and Oceania. Temperature of the coldest season, seasonal temperature differences, and rainfall in the coldest season are the key factors controlling its spread. Under future climate scenarios, the total suitable area for this pathogen is expected to expand and shift toward higher latitudes (northward). Its distribution center will move northeast from Tamanrasset Province in Algeria by up to 960 km. These predictions can help authorities develop early monitoring and control measures, thereby reducing potential harm to forestry and natural ecosystems.

## 1. Introduction

The dual drivers of globalization and climate change are profoundly reshaping the distribution boundaries and ecological dynamics of biological populations [1]. Against this backdrop, plant pathogenic organisms, especially those with broad host adaptability and environmental tolerance, face increasingly heightened risks of cross-border transmission and colonization, posing a significant threat to global forestry security, the stability of natural ecosystems, and biodiversity conservation [2,3]. Within this context, oomycetes—particularly members of the genus *Phytophthora* and its closely related taxa—have emerged as especially damaging invaders, capable of causing rapid and widespread forest declines. Tree diseases caused by *Phytophthor* aspecies pose a significant threat to global agricultural production due to their rapid outbreak, severe damage, and considerable challenges in management [4]. Among them, *Phytopythium litorale* (Nechwatal & Mendgen, 2006), as a newly identified pathogenic species, is attracting increasing attention in the fields of plant pathology and biosecurity. Although *P. litorale* shares many ecological traits with better-studied Phytophthora species, its global distribution and invasion potential remain largely unexplored. Since its first isolation from the rhizosphere of reeds along the shores of Lake Constance in Germany [5], *P. litorale* has been successively reported on various host plants in multiple regions including Scotland [6], Turkey [7], Iran [8], China [9], and the United States [10]. Studies indicate that *P. litorale* is not only widely distributed in aquatic environments such as wetlands and rivers, but is also capable of infecting economically and ecologically important plants including rhododendron, red raspberry, kiwifruit, and apple [11]. However, existing studies have been largely regional, focusing on local occurrence records or limited host ranges, and a systematic, global-scale assessment of its potential distribution and future invasion risks remains absent. This knowledge gap critically hinders the development of proactive quarantine measures and cross-border risk assessments. Delineating the ecological distribution range of *P. litorale* is therefore of great significance for preventing the economic losses and ecological risks associated with this pathogen.

Species distribution models have become a central tool in ecology and biogeography for predicting the potential geographic distribution of species [12]. Among these, the MaxEnt model is widely recognized as one of the most effective and is the most frequently used in various species distribution studies [13,14]. By integrating known species occurrence data (longitude and latitude) with corresponding environmental variables, the MaxEnt model compares climatic similarity between locations where species are present and other areas within the study region, thereby predicting the potential distribution range and habitat suitability of the species. Compared with other models, MaxEnt demonstrates higher predictive accuracy and can yield reliable results even with imprecise occurrence records [15,16,17]. Particularly excelling in handling small sample sizes and complex variable interactions, the maximum entropy model has been successfully applied to predict the distribution and climate change responses of various plant diseases, such as cabbage Verticillium wilt [18], pine wilt disease [19], and pitch canker [20]. For *P. litorale*, the current number of publicly available occurrence records is limited, and the environmental variables are known to interact in complex ways (e.g., temperature, precipitation). These characteristics make MaxEnt particularly suitable for this study, as it can produce robust predictions from small sample datasets and efficiently handle multicollinearity and interaction effects among bioclimatic variables without requiring absence data, which is a major advantage over many traditional modeling approaches. Nevertheless, there remains limited research on the future distribution trends and ecological adaptation mechanisms of *P. litorale* under changing climatic scenarios.

Based on global distribution data of *P. litorale*, this study employed the MaxEnt model to simulate and predict the dynamics of its potential suitable habitats under current climatic conditions as well as under future SSP1-2.6, SSP3-7.0, and SSP5-8.5 scenarios, and further analyzed the shift trajectory of its distribution centroid. The novelty of this study lies in: (1) being the first global-scale prediction of *P. litorale* potential distribution, moving beyond previous regional occurrence reports; (2) the use of systematic parameter optimization using ENMeval to avoid model overfitting, which has not been applied to this species before; and (3) projection under three future climate scenarios to identify climate-driven range shifts and centroid migrations, thereby offering actionable insights for climate-adaptive biosecurity planning. The findings are expected to provide a theoretical basis for understanding the global distribution pattern of this pathogen, and for developing early monitoring, warning, and regional prevention and control strategies.

## 2. Materials and Methods

### 2.1. Data Sources and Processing

This study utilized databases including the Global Biodiversity Information Facility (GBIF) (accessed on 20 October 2025), CABI Digital Library (https://www.cabi.org, accessed on 20 October 2025), and EPPO (accessed on 20 October 2025), along with a review of publicly available domestic and international journals and papers (accessed on 20 October 2025), to collect global distribution records of the pathogen. The obtained occurrence coordinates were compiled in Excel and subsequently imported into ArcGIS software (version 10.8). To ensure data quality, a buffer zone analysis method with a 10 km distance threshold was applied in ArcGIS to screen the distribution points, removing records with missing or invalid coordinates and duplicate entries. A buffer zone analysis method was applied to screen the distribution points, removing erroneous and duplicate records. After screening and deduplication, 150 distribution records of *P. litorale* retained for use in this study. These records were organized in Excel by species, longitude, and latitude, and saved in CSV format according to the input requirements of the MaxEnt software (version 3.4.4) for further analysis. To reduce sampling bias, spatial thinning was applied using a 10 km × 10 km grid cell via the spThin R package (version 0.2.0) with 100 iterations, minimizing the effects of uneven sampling effort across the species’ range. This thinning process retained all 150 records as unique geographic locations (i.e., no two points fell within the same 10 km grid cell). The thinned occurrence records were then used to guide background sampling in MaxEnt, with 10,000 background points randomly selected from the study area.

### 2.2. Environmental Variables

The climatic data were obtained from the WorldClim database (https://worldclim.org accessed on 25 October 2025), including historical climate data (1970–2000) and future climate projections (2021–2100). The historical climate dataset comprised 19 bioclimatic variables (Bio1-Bio19), selected to reflect annual trends, seasonality, and limiting environmental factors. Soil data were sourced from the Harmonized World Soil Database (HWSD) grid dataset provided by the Food and Agriculture Organization of the United Nations (http://www.fao.org/soils-portal accessed on 25 October 2025) [21]. Topographic data included elevation, slope, and aspect. A total of 36 environmental variables were compiled (Table 1). Future climate projections were derived from the BCC-CSM2-MR climate model under the Coupled Model Intercomparison Project Phase 6 (CMIP6). This model was selected because it demonstrates high accuracy and reliability in simulating the East Asian summer monsoon and associated precipitation and temperature patterns over China [22], and it has been widely applied in species distribution studies for Chinese region. Three future climate scenarios defined by Shared Socioeconomic Pathways (SSPs) were selected for projection: SSP1-2.6 (low radiative forcing scenario), SSP3-7.0 (medium to high forcing scenario), and SSP5-8.5 (high forcing scenario) [23,24]. All environmental layers were resampled to a common grid (resolution = 30 arc-seconds; CRS = WGS84). Continuous variables were resampled using bilinear interpolation and categorical variables using nearest-neighbor. Layers were aligned and masked to the calibration area prior to modeling. To mitigate multicollinearity and avoid model overfitting, Pearson correlation analysis was performed on the 36 environmental variables. Highly correlated variable pairs (|r| > 0.8) were identified, and the variable with lower permutation importance in preliminary MaxEnt models was removed [25]. Variable importance was further assessed using the Jackknife test to ensure retention of ecologically meaningful predictors. All environmental variables were selected based on documented effects of temperature, precipitation, soil properties, and terrain on the growth, survival, and distribution of *Phytopythium* and related oomycete species [26,27,28,29]. Terrain variables were included as proxies for microclimate and soil moisture conditions.

### 2.3. Selection of Key Environmental Variables

To accurately identify the key environmental variables, this study first imported the collected 150 distribution records of *P. litorale* and 36 ecological factors into MaxEnt 3.4.4 for preliminary analysis. To avoid model overfitting caused by highly correlated environmental variables, a multicollinearity analysis was conducted for variable screening. Using ArcGIS, 10,000 points were randomly generated within the study area, and the values of each environmental variable at these points were extracted. Pearson’s correlation coefficient matrix and variance inflation factor (VIF) were calculated for all variable pairs. Variables with |R| > 0.8 or VIF > 10 were flagged as highly correlated. For each flagged variable pair, the variable with lower ecological relevance to the life history of *P. litorale* was excluded, while the variable with clear biological effects was retained [30]. When ecological relevance was comparable, priority was given to retaining the variable with a lower VIF or higher contribution in univariate models. Based on this screening, the non-collinear variables were retained for subsequent modeling. Following the collinearity screening, the retained subset of 12 variables was used to formally construct the MaxEnt model. In the model configuration, 75% of the species occurrence points were randomly selected as the training set, and the remaining 25% were used as the test set, with the process repeated 10 times. After model execution, the contribution of each environmental factor was analyzed based on the Jackknife test and permutation importance results to identify the dominant environmental factors influencing the current distribution of *P. littorale* [31]. It should be noted that the contribution results were solely used for model interpretation and were not involved in the prior variable screening process, so as to avoid issues of circular dependency.

### 2.4. Result Output and Model Accuracy Verification

To prevent overfitting due to excessive model complexity and to avoid subjective parameter selection, this study systematically optimized the model parameters using the ENMeval package (version 2.0) on the R 4.3.2 platform (version 4.3.2) [32]. By evaluating the combined effects of different feature classes—including hinge (H); linear (L); linear and quadratic (LQ); linear, quadratic and hinge (LQH); linear, quadratic, hinge and product (LQHP); and all feature types (LQHPT) and regularization multipliers ranging from 0 to 6 at intervals of 0.5 [33]—the optimal parameter set was selected based on the lowest Akaike information criterion value and subsequently used to build the final model. To fully assess model generalization and potential overfitting, we calculated the cross-validated AUC (mean ± standard deviation across folds) separately for training and test data under all climate scenarios and time periods. These results are presented in Supplementary Table S1.

During model calibration, the calibration area (M) was rigorously defined as the set of geographic areas accessible to *P. litorale*, considering its freshwater-dependent ecology and dispersal limitations; thus, M encompassed all global regions excluding oceans and permanent ice sheets, including both terrestrial areas and inland water bodies (lakes and rivers), based on the ecological characteristics of *P. litorale* distributed in freshwater habitats, to comprehensively cover the species’ potential habitat. Background points were sampled exclusively from M after excluding areas with permanent ice sheets and oceans, ensuring they represent the environmental conditions accessible to the species. We uniformly sampled 10,000 background points within M, using a consistent land–inland water mask for current and future climate projections. All runs were performed with a fixed random seed (seed = 12,345) to ensure reproducibility.

Based on the known species distribution data, we selected the optimal threshold from the MaxEnt model output file “maxentResults.csv” for distribution area validation. The model prediction results (in ASC format) were converted into spatial distribution maps using ArcMap software (version 10.8). To comprehensively evaluate the accuracy and robustness of the model predictions, the Receiver Operating Characteristic curve analysis was applied, and the model performance was assessed by calculating the Area Under the Curve value. The evaluation criteria for AUC values are as follows: 0.5–0.6 indicates prediction failure; 0.6–0.7 represents poor prediction; 0.7–0.8 denotes moderate prediction; 0.8–0.9 signifies good prediction; and 0.9–1.0 indicates excellent prediction performance [34].

### 2.5. Changes in the Area of Potential Suitable Areas and Shifts in the Center of Gravity

To accurately display the spatial distribution pattern of the potential suitable areas for *P. litorale*, the modeling results from MaxEnt were imported into ArcGIS software. Using the Natural Breaks method in the reclassification tool, the disease occurrence suitability was classified into four grades from low to high: non-suitable area, low-suitable area, moderately suitable area, and highly suitable area [20]. This classification was used to map the global potential suitable areas for *P. litorale* under both current and future climate scenarios. For further quantitative analysis, we converted continuous suitability to binary suitable/unsuitable using the “10 percentile training presence” threshold (value = 0.344), which was selected because it excludes the lowest 10% of predicted suitability values at training presence locations, thereby reducing the influence of potential occurrence record errors or outliers while maintaining a conservative estimate of suitable habitat. Area change and centroid analyses were conducted based on this binary map to ensure comparability across scenarios and periods. On this basis, the area of potential suitable regions under different climate scenarios for each period was calculated uniformly. Additionally, the centroid coordinates of the suitable areas were computed using the Mean Center tool in the SDM Toolbox, and the migration trajectory was plotted accordingly.

Centroid coordinates were calculated based on the predicted suitable habitat distribution rasters under different climate scenarios. First, the continuous suitability rasters output by the MaxEnt model were converted into binary suitable habitat distribution maps using the threshold determined by the Maximum Youden Index. An area field was added to the attribute table, and the Raster Calculator tool was then used to calculate the area corresponding to each suitable habitat class based on pixel size. On this basis, the geographic centroids of suitable habitats under different climate scenarios were calculated to analyze the geographic shift in centroids. A workflow diagram summarizing the entire methodology is presented (Figure S1).

## 3. Results

### 3.1. Model Accuracy

**Figure 1:**
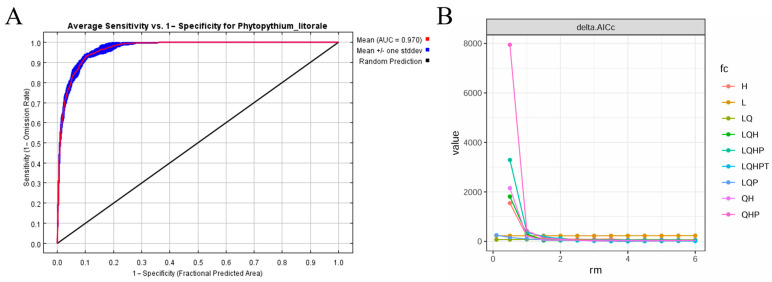
ROC analysis and delta AICc changes in the distribution prediction model for *P. littorale*. (**A**) ROC curve for the potential distribution prediction of *P. littorale*. (**B**) The relationship curve between the delta AICc value of the model and the rm value.

The MaxEnt model, constructed after systematic parameter optimization (RM = 4, FC = LQHPT, ΔAICc = 0 for the best model), achieved high standards in both statistical performance and ecological rationality (Figure 1B). Model performance was evaluated by cross-validation. As shown in Supplementary Table S1, training AUC values were consistently high (≥0.95) across all scenarios, while test AUC values ranged from 0.89 to 0.93 with standard deviations generally below 0.03 (except for SSP3-7.0 2081-100, where the test AUC SD was 0.259, see Table S1). All test AUC values were significantly higher than that of a random model (0.5), indicating a high level of simulation performance and reliable experimental results. Nevertheless, AUC interpretation has known limitations: it is sensitive to the choice of background points and does not directly reflect spatial prediction bias; therefore, the excellent AUC value should be considered alongside other model evaluation metrics.

### 3.2. Analysis of Environmental Factors

This study employed 36 bioclimatic variables combined with the MaxEnt model to predict the global potential distribution of *P. littorale*. MaxEnt outputs were interpreted as relative habitat suitability rather than true occurrence probability. Twelve significant factors were ultimately identified through Pearson correlation analysis, namely Bio2, Bio3, Bio11, Bio14, Bio19, Aspect, Slope, Elevation, T_cec-clay, T_GRAVEL, T_silt, and T_texture. The importance contribution values of each environmental variable are presented (Table 2, Figure S2). The contribution rates of the twelve selected environmental variables during modeling were all greater than zero, indicating that no irrelevant variables were included in the simulation. Moreover, the key environmental variables collectively contributed 73.6% to the overall model, with Bio11 exhibiting the highest contribution rate. This demonstrates that the mean temperature of the coldest quarter exerts the greatest influence on the distribution of *P. litorale*. Suitable ranges of key variables were summarized from pixels classified as suitable under the selected threshold.

**Figure 2:**
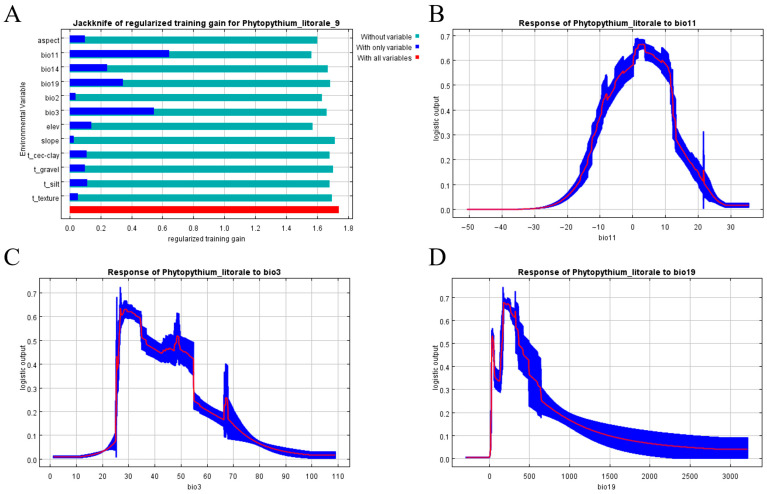
The results of the k-tile analysis and response curves of the key environmental variables that dominate the changes in the suitable habitat area of *P. littorale*. (**A**) Knife-cutting analysis diagram. (**B**) Mean Temperature of Coldest Quarter. (**C**) Isothermality. (**D**) Precipitation of coldest quarter.

The results of the jackknife analysis for the environmental variables of *P. litorale* are shown (Figure 2). The regularized training gain algorithm takes into account the interdependence among variables, allowing for standardized comparison of the contributions of predictors. When using a single environmental factor, the three factors with the greatest impact on the regularized training gain were Bio11, Bio3, and Bio19. These three indicators were selected for further analysis, and the response curves between each environmental variable and the relative suitability of *P. litorale* are presented (Figure 2). Based on the response curves of the environmental variables, the range of values influencing the relative habitat suitability of *P. litorale* presence can be determined. A relative suitability index greater than 0.5 (used here as a relative threshold) indicates environmental conditions suitable for the species. The suitable ranges are: mean temperature of the coldest quarter, −5.52 to 11.52 °C; isothermality, 33.4 to 36.53% (Bio3 is a ratio, expressed as a percentage); and precipitation of the coldest quarter, 160.24 to 363.75 mm (Figure 2). These parameter ranges provide a suitable probability of survival for *P. litorale*.

### 3.3. Distribution of P. litorale in Its Suitable Habitats Under Current Climatic Conditions

**Figure 3:**
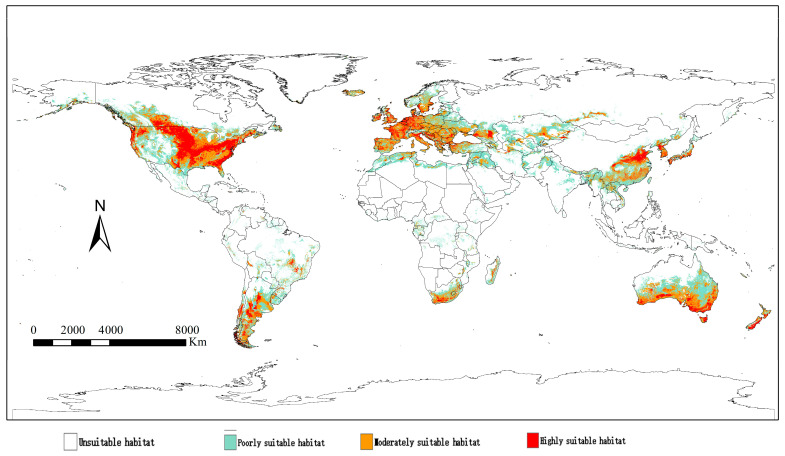
Prediction of the suitable habitats of *P. litorale* worldwide under the current climate conditions.

Using ArcGIS software, a map of the suitable areas for *P. litorale* current climatic conditions was generated (Figure 3), and the areas occupied by different suitability levels are summarized (Table 3). The suitable habitats for *P. litorale* are primarily distributed in North America, Europe, Asia, Oceania, and other regions. The total suitable area amounts to 3702.85 × 10^4^ km^2^, while the highly suitable area is relatively small, measuring only 614.01 × 10^4^ km^2^.

### 3.4. Distribution of P. litorale in Its Suitable Habitats Under Future Climate Conditions

**Figure 4:**
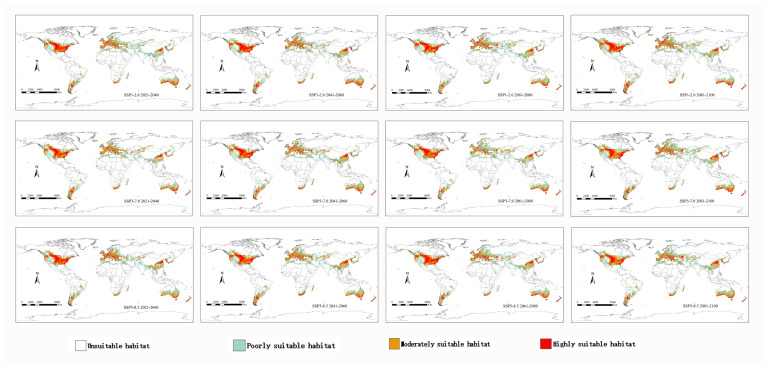
Prediction of the suitable habitats of *P. litorale* in the world under different future climate change scenarios.

Projected future climate scenarios are presented (Figure 4), which illustrates differentiated spatiotemporal evolution patterns of suitable habitats under varying emission pathways. Across the three future climate scenarios, the total suitable area for *P. litorale* was projected to increase overall, with particularly notable changes in the highly suitable zones (Table 3). Simulation outcomes reveal significant variations across scenarios and time periods: under the SSP3-7.0 scenario, the total suitable area reaches its maximum during 2081–2100 (an increase of approximately 4.8% relative to current), while the smallest total area occurs during 2021–2040 (a decrease of approximately 5.8%). Regarding highly suitable areas, the largest extent occurs under SSP1-2.6 during 2081–2100 (an increase of approximately 7.7%), whereas the smallest occurs under SSP3-7.0 during 2061–2080 (a decrease of approximately 14.2%). Spatial analysis indicates that the distribution patterns of potential low to moderately suitable habitats for *P. litorale* future climate conditions generally align with current trends. However, under SSP3-7.0 (2061–2080), a pronounced contraction in highly suitable areas is evident. Additionally, over time, the spatial distribution of suitable habitats exhibits a discernible latitudinal shift.

### 3.5. Prediction of the Distribution Range of P. litorale in Global Suitable Areas Under Future Climate Conditions

**Figure 5:**
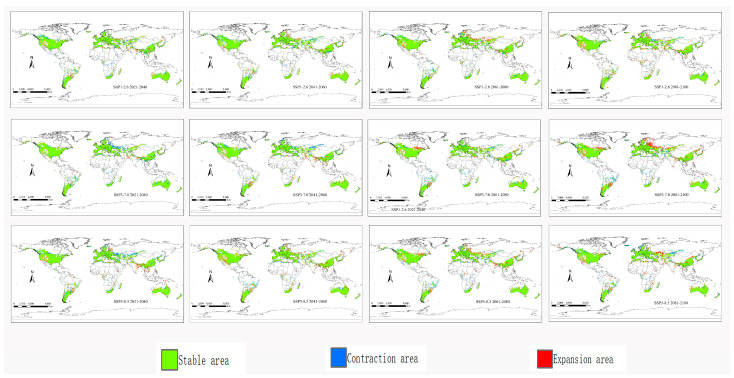
Spatiotemporal dynamics of potential suitable habitats for *P. litorale* under climate change scenarios.

This study elucidates the potential distribution dynamics of *P. litorale* future climate change scenarios through geospatial analysis, providing a scientific basis for the future prevention and control of this pathogen. Ecologically, these shifts imply that areas of habitat gain may face increased pathogen introduction risk, while stable core regions could serve as refugia requiring long-term monitoring. The results indicate that the expansion rate of the suitable habitat area for *P. litorale* is greatest under the SSP3-7.0 scenario (2081–2100), with an increase of 14.79% compared to the current climate conditions (Table 4; Figure 5). Approximately 547.72 × 10^4^ km^2^ of currently unsuitable area is projected to become suitable, primarily in North America (California, USA; northern Mexico), Europe (Ukraine, western Russia, Finland, Norway), and Asia (eastern China, northern India, Turkey, Iran, Kazakhstan). In contrast, the largest contraction of suitable area occurs under the SSP3-7.0 scenario (2021–2040), showing a reduction of 12.83% relative to the current climate, mainly concentrated in Kazakhstan, Belarus, Ukraine, and neighboring regions. Overall, the changes in suitable areas for *P. litorale* exhibit similar spatial patterns over time. Under the SSP1-2.6 pathway, both contraction and expansion ratios are relatively low, resulting in the most stable spatial pattern, whereas the SSP3-7.0 pathway drives the most pronounced shifts. These findings suggest that climate change may drive significant geographical shifts rather than merely simple area changes in the suitable distribution of *P. litorale*, with spatial restructuring being a key characteristic of future habitat suitability dynamics.

### 3.6. Migration of the Center of P. litorale Under Future Climate Conditions

**Figure 6:**
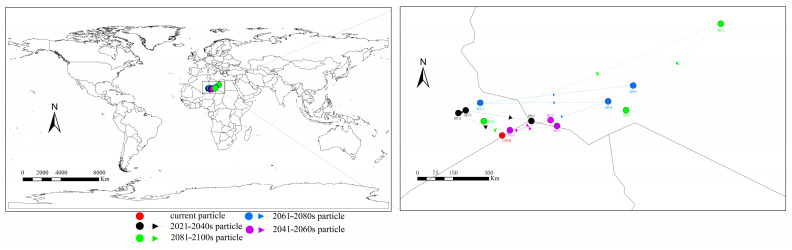
Migration of the ecological center of the lake-edge *P. litorale* under different climate scenarios.

Spatiotemporal analyses indicate that under different climatic scenarios, the centroid of the habitat suitable for *P. litorale* exhibits distinct geographical shifts, with an overall northeastward movement trend. Specific distribution patterns and changes are shown (Figure 6). Currently, the suitable centroid for *P. litorale* is located in Tamanrasset Province, Algeria (22.96244° N, 10.960572° E). Given the inherent uncertainties in species distribution modeling, the reported centroid coordinates should be interpreted as approximate locations rather than exact points. During the 2081–2100 period, the centroid shift is most pronounced. Under the SSP1-2.6 scenario (2081–2100), the centroid of suitable habitat moves northwestward but remains within Tamanrasset Province, Algeria (23.504002° N, 10.262228° E), with a displacement of approximately 93.6 km. Under the SSP3-7.0 scenario (2081–2100), the centroid shifts to Ghat, Ghat District, southwestern Libya (27.208274° N, 19.241982° E), covering a distance of approximately 960 km. Under the SSP5-8.5 scenario (2081–2100), the centroid relocates to Sabha, Sabha District, Libya (23.922035° N, 15.652635° E), with a displacement of approximately 490.62 km.

In contrast, during the 2041–2060 period under SSP1-2.6, SSP3-7.0, and SSP5-8.5 scenarios, the centroid shifts are relatively small and consistently northeastward. The centroids are situated in Sabha, Sabha District, Libya (23.545711° N, 12.794886° E); near the Algeria–Niger border (23.161435° N, 11.25339° E); and near the Libya–Niger border (23.32372° N, 13.03837° E), respectively. The predicted distances from the current suitable centroid are approximately 198.4 km, 37.42 km, and 216.5 km.

In summary, across both the 2041–2060 and 2081–2100 projections, the centroid of suitable habitat for *P. litorale* generally shows a progressive northeastward displacement in all temporal forecasts.

## 4. Discussion

Following the recommendations of Phillips et al., this study employed the ENMeval 2.0 package to systematically optimize the parameters of the MaxEnt model, thereby mitigating potential model bias resulting from reliance on default settings [35]. The optimal parameter combination was identified as RM = 4 and FC = LQHPT, under which the corrected Akaike Information Criterion (AICc) value of the model reached 0. This outcome underscores the importance of parameter optimization in constructing robust ecological niche models. Furthermore, validation results (Figure 1) demonstrate that under current climatic conditions, the model achieved an AUC value exceeding 0.95, indicating strong discriminatory ability and predictive stability, though it is important to note that AUC values can be inflated by spatial autocorrelation and biased background sampling [36]. These findings align with AUC results reported in similar distribution modeling studies for pathogens such as *Phytophthora nicotianae* [37].

The jackknife test and response curve analysis in this study indicate that Bio11, Bio3, and Bio19 are primary environmental variables influencing the potential distribution pattern of *P. litorale*. Mechanistically, the mean temperature of the coldest quarter (Bio11) likely acts by limiting mycelial growth and overwintering survival; temperatures below −5.52 °C may suppress hyphal extension and spore germination, while temperatures above 11.52 °C during the coldest period could disrupt normal dormancy–activity cycles. Isothermality (Bio3) reflects the stability of diurnal temperature variation; the narrow suitable range (33.4–6.53%) suggests that the species requires relatively stable temperature fluctuations to support zoospore development and host infection. Precipitation of the coldest quarter (Bio19) directly influences soil water availability; the identified range (160–64 mm) likely ensures sufficient soil moisture for zoospore motility and infection while avoiding waterlogged conditions that might suppress host root respiration. This finding is consistent with the general principles of oomycete ecological adaptation. MaxEnt outputs were interpreted as relative habitat suitability rather than true occurrence probability. Suitable ranges of key variables were summarized from pixels classified as suitable under the selected threshold. Multiple previous studies have highlighted that water availability is a key factor influencing the distribution, diversity, and abundance of soil oomycetes [38]. High-humidity environments significantly enhance oomycete dispersal and colonization success by promoting sporangium release, zoospore motility, and the formation of asexual reproductive structures [39]. In the present study, *P. litorale* exhibited a higher probability of presence within a precipitation range of approximately 160.24 to 363.75 mm during the coldest quarter. This moisture range likely provides the necessary liquid water film for zoospore activity and host infection, which is consistent with the report by Zhang et al. regarding the activity of the oomycete *P. litorale* under moderate precipitation conditions [40].

Temperature is also a critical factor regulating the life activities of oomycetes [27]. The response curve predicted in this study indicates that *P. litorale* shows a relatively high survival probability at mean temperatures of the coldest quarter ranging from −5.52 °C to 11.52 °C, suggesting a certain degree of adaptation to low temperatures. Regarding low-temperature tolerance, this adaptation is consistent with the general capacity of many oomycetes to remain active near freezing temperatures, a trait commonly observed in various *Phytophthora* and *Pythium* species. This aligns with the findings of Scagel et al. on three major *Phytophthora* species (*P. cinnamomi*, *P. pini*, and *P. plurivora*) in the Pacific Northwest region [41], which reported significant differences in minimum growth temperatures and low-temperature lethal thresholds among strains, with some isolates capable of surviving at 0 °C or even −5 °C. Additionally, isothermality (Bio3) in the range of 33.4% to 36.53% was found to be suitable for the species, reflecting its sensitivity to seasonal temperature variation. This is consistent with the requirement of temperature stability for oomycete survival, a pattern also observed by Ke et al. in grassland ecosystems in China, where temperature seasonality significantly influenced the abundance distribution of pathogenic oomycetes [42]. Apart from climatic factors, topographic and soil conditions also affect the potential suitable habitats of this pathogen. Topographic features such as elevation and slope alter local hydrothermal conditions and surface runoff patterns, thereby influencing water retention and microclimate formation, which in turn constrain the survival and dispersal environments of the pathogen [43]. Meanwhile, soil properties including organic matter content and drainage capacity directly affect the growth and root health of host plants. They also indirectly influence the survival, infectivity, and dispersal potential of soil-borne pathogens by regulating soil moisture, aeration, and microbial community structure [44,45]. Consequently, even within climatically suitable broad regions, the actual distribution of the pathogen tends to exhibit a localized, patchy pattern determined by site-specific topographic and soil characteristics. Quantitatively, based on the percent contribution rates in Table 2, climatic factors (Bio11, Bio3, Bio14, Bio19, Bio2) collectively account for 76.2% of the total contribution to the model, whereas soil-related factors (T_GRAVEL, T_cec-clay, T_silt, T_texture) account for only 8.9%. Topographic factors (elevation, aspect, slope) account for the remaining 14.9%. However, it is important to note that while high-resolution climatic data are available for future scenarios (e.g., SSPs), soil data at the same spatial and temporal resolution are generally not available for future periods. In practice, soil properties are treated as static in species distribution modeling due to the lack of dynamically projected soil layers under future climate scenarios. This constitutes a clear limitation of the current study. Further investigation is needed to elucidate the overall mechanisms by which temperature, precipitation, topography, and soil interactively regulate the potential distribution of this pathogen.

This study comprehensively applied ecological niche modeling and multi-scenario climate projections to systematically evaluate potential shifts in the geographical distribution of *P. litorale* from the present to the end of the 21st century. Under current climatic conditions, the model predicts that areas of high suitability for this pathogen are primarily located in North America, Europe, East Asia, Oceania, and South Africa, which aligns with previously reported occurrence records and its water-borne biological characteristics [46,47]. Notably, regions such as Argentina and New Zealand are also identified as highly suitable, yet no formal disease reports have been documented in these areas to date, suggesting possible surveillance gaps or latent risks. A major limitation of this study is the use of only one GCM (BCC-CSM2-MR), which does not account for structural uncertainties across different climate models.

The distribution of this species exhibits distinct spatiotemporal heterogeneity under different Shared Socioeconomic Pathways (SSPs). Under the sustainable pathway (SSP1-2.6), the total suitable area for *P. litorale* projected to increase slightly by the end of the century (approximately 3.2%), with a corresponding expansion of highly suitable regions, indicating a potential shift toward higher latitudes and altitudes under moderate warming. The core distribution area remains highly stable, with over 80% of its extent continuing to provide suitable habitat. From a biological perspective, the moderate expansion under SSP1-2.6 suggests that the pathogen could gradually extend its range into previously cooler regions, potentially increasing disease pressure on naive host populations in boreal and alpine ecosystems. The stability of the core area implies that established hotspots (e.g., western Europe, eastern China) will remain persistently suitable, requiring continuous surveillance.

However, under medium-to-high-emission (SSP3-7.0) and high-emission (SSP5-8.5) scenarios, distribution volatility increases markedly. Especially under SSP5-8.5, the area of highly suitable habitat is projected to decline by about 11.1% by the century’s end. Ecologically, this contraction in highly suitable areas does not necessarily imply reduced risk; instead, it may reflect a “habitat compression” effect, where climate becomes too extreme for optimal growth but the pathogen persists in refugia with higher local moisture or topographic buffering. This aligns with the theoretical mechanism that intense warming under high-forcing scenarios could potentially approach or even exceed the species’ climatic tolerance limits based on current physiological understanding, which would lead to contraction of suitable habitat [48], and corresponds with the nonlinear fluctuation pattern of habitat shrinkage observed by Zhang et al. in simulations of *Phytophthora cinnamomi* distribution under SSP5-8.5 [40]. Spatially, a “northward and upward” shift pattern emerges: expansion occurs mainly in higher-latitude and higher-altitude zones (e.g., northern Canada, Northern Europe, Siberia, and northeastern China), while contraction concentrates along the southern margins and lower-elevation areas of the current distribution, consistent with distributional shifts observed for many temperate species under climate change [49]. Biologically, this shift implies that high-latitude regions with currently cold winters may become newly suitable for overwintering, while low-latitude populations may experience increased heat stress, potentially altering the timing of sporulation and host infection windows.

Regarding the centroid coordinate reported in the Results, a necessary clarification is needed: this centroid is a mathematical center (i.e., geometric center) calculated from the predicted suitability raster. It reflects the overall spatial distribution trend of suitable habitats across the entire study area, but does not necessarily indicate that the coordinate point itself has high habitat suitability. When suitable habitats are fragmented (e.g., in parts of the Sahara region in this study), the centroid may fall in areas with low or even no actual suitability. Therefore, this coordinate should be interpreted as a spatial statistic rather than the central point of the species’ actual distribution.

Limitations of this study include the following: (1) human-mediated dispersal pathways such as international trade of seedlings and soil-attached commodities have not been incorporated, which may lead to underestimation of long-distance dispersal risks; (2) the analysis relies on the assumption of climatic niche conservatism and does not account for potential adaptive evolution of the pathogen—thermal adaptation experiments across multiple generations would be needed to assess this; (3) we did not assess extrapolation risks associated with predictions under novel climate conditions, which may affect the reliability of projections in regions where future climates deviate substantially from current training data; (4) our projections are based on a single global climate model (BCC-CSM2-MR) from the CMIP6 framework, which may not fully capture the range of uncertainties associated with different climate model structures; (5) the occurrence records used may contain sampling bias, as most reports come from a few countries (e.g., China, Turkey, USA), potentially underrepresenting tropical and southern hemisphere regions. Future studies should integrate occurrence data from citizen science platforms and targeted field surveys to reduce geographic bias. (6) We also acknowledge the lack of independent validation data; because all occurrence records and the random 25% test split come from the same GBIF/CABI sources, spatial autocorrelation may lead to slightly overoptimistic metrics, though the impact on our relative suitability patterns and centroid shifts is likely limited given the sample size constraints. The present findings can directly assist in the future management of *P. litorale* under climate change by identifying high-risk regions for targeted surveillance, informing the revision of plant quarantine lists, and guiding the allocation of monitoring resources under different climate scenarios (SSP1-2.6, SSP3-7.0, SSP5-8.5). Specifically, the projected northward and upward shifts suggest that current low-risk areas at higher latitudes may become suitable, warranting proactive monitoring, while contracting low-latitude areas could allow for relaxed management, enabling a dynamic, risk-based adaptation strategy. Recommendations for further research are: (1) developing dispersal network models using global port-inspection data; (2) quantifying thermal adaptation potential of strains through cultivation experiments across temperature gradients; (3) monitoring early-stress symptoms in crops within high-risk areas via UAV-based multispectral remote sensing; (4) incorporating multiple GCMs and ensemble forecasting approaches in future studies to better account for climate model uncertainty; and (5) conducting multivariate environmental similarity surface or Mahalanobis distance analyses to identify regions with novel climates where extrapolation should be interpreted with caution. The findings provide a quantitative basis for updating plant quarantine lists and formulating region-specific management strategies, offering important early-warning insights for safeguarding forestry security and ecological stability.

## 5. Conclusions

Simulations based on the MaxEnt model under global climate-change scenarios reveal spatiotemporal changes in the potential geographic distribution of *P. litorale*. Under the current climatic baseline, highly suitable areas are mainly located in Europe, Oceania, and North America, with distribution patterns constrained jointly by temperature and precipitation. Across future climate scenarios, suitable ranges generally expand toward higher latitudes and elevations. Under the high-emission scenario SSP5-8.5, the total suitable area is projected to increase by approximately 3% by the end of the 21st century; however, highly suitable regions are expected to contract along the southern edges of the current distribution belt, forming a spatial restructuring pattern characterized by “northward expansion and southern contraction”. Distribution-dynamic analysis indicates that stable areas are concentrated in the present core distribution zone, while expansion zones largely overlap with emerging habitats such as the tundra-forest transition south of the Arctic Circle, coastal wetlands, and mountainous wetlands in Scandinavia. These shifts may create new niche opportunities for the species and could entail potential risks to wetland ecosystems via biological invasion pathways. The results highlight the need to establish early-monitoring systems in expansion hotspots and underscore that future studies should integrate physiological-tolerance experiments and human-mediated dispersal models to improve prediction accuracy. In summary, the geographic distribution of *P. litorale* exhibits a sensitive and complex response to climate change, and its ongoing dynamics will pose continuing challenges to biosecurity and integrated management of coastal ecosystems.

## Figures and Tables

**Table 1:** Environmental Factor Data Information.

Index	Factor	Description
Temperature	Bio1	Annual Mean Temperature
	Bio2	Mean diurnal range
	Bio3	Isothermality
	Bio4	Temperature seasonality
	Bio5	Max Temperature of Warmest Month
	Bio6	Min Temperature of Coldest Month
	Bio7	Temperature Annual Range
	Bio8	Mean Temperature of Wettest Quarter
	Bio9	Mean Temperature of Driest Quarter
	Bio10	Mean Temperature of Warmest Quarter
	Bio11	Mean Temperature of Coldest Quarter
Precipitation	Bio12	Annual Precipitation
	Bio13	Precipitation of Wettest Month
	Bio14	Precipitation of Driest Month
	Bio15	Precipitation Seasonality
	Bio16	Precipitation of Wettest Quarter
	Bio17	Precipitation of Driest quarter
	Bio18	Precipitation of Warmest Quarter
	Bio19	Precipitation of Coldest Quarter
Terrain	Asp	Aspect
	Slo	Slope
	Alt	Elevation
Soil	T_bulk	Topsoil bulk density
	T_cec_soil	Topsoil CEC
	T_cec_clay	CEC of the clay fraction
	T_clay	Topsoil Clay Fraction
	T_ece	Topsoil Salinity
	T_esp	Topsoil Sodicity
	T_gravel	Topsoil Gravel Content
	T_oc	Topsoil Organic Carbon
	T_ph	Topsoil ph
	T_ref_bulk	Topsoil Reference Bulk Density
	T_sand	Topsoil Sand Fraction
	T_silt	Topsoil Silt Fraction
	T_usda_texture	Topsoil USDA Texture
	T_usda	Topsoil USDA Texture Class

**Table 2:** Contribution rates and permutation importance values of dominant environmental factors affecting the distribution of *P. littorale*.

Code	Environmental Factors	Percent Contribution/%	Permutation Importance/%
1	Bio11	25.1	39.3
2	Bio3	23.8	19.1
3	Bio14	12.8	3.6
4	Bio19	11.9	7
5	Elevation	8.9	11.2
6	Aspect	3.8	3
7	T_GRAVEL	2.9	3.7
8	T_cec-clay	2.8	3.1
9	Bio2	2.6	4.1
10	T_silt	2.4	3.3
11	Slope	2.2	1
12	T_texture	0.8	1.8

**Table 3:** Area of Suitable Regions for *P. litorale* under Different Climate Scenarios (10,000 square kilometers).

Climate	Period	Poorly	Moderately	Highly	Total
		Suitable Habitat	Suitable Habitat	Suitable Habitat	Suitable Area
current	1971–2000	1866.55	1222.29	614.01	3702.85
SSP1-2.6	2021–2041	1942.52	1134.17	573.26	3649.95
	2041–2060	1894.56	1153.54	621.02	3669.12
	2061–2080	1956.73	1198.62	583.98	3739.33
	2081–2100	1886.1	1274.57	661.38	3822.05
SSP3-7.0	2021–2040	1730.31	1194.55	564.3	3489.16
	2041–2060	1804.77	1226.56	636.53	3667.86
	2061–2080	1855.59	1162.83	526.83	3545.25
	2081–2100	1971.22	1277.04	632.38	3880.64
SSP5-8.5	2021–2040	1796.64	1187.52	619.86	3604.02
	2041–2060	1838.83	1281.99	608.43	3729.25
	2061–2080	1966.19	1214	616.5	3796.69
	2081–2100	2054.76	1208.72	551.36	3814.84

**Table 4:** Changes in the distribution areas of *P. litorale* in different periods and under different scenarios.

Period	Climate Scenario	Habitat Area	Stable Area	Contraction Area	Expansion Area	Net Change *	Contraction Area	Expansion Area
						(%)	(%)	(%)
Current	-	3702.85						
2021–2040	SSP1-2.6	3649.95	3054.71	410.91	275.99	3.64	11.1	7.45
	SSP3-7.0	3489.16	2990.02	475.01	229.03	6.64	12.83	6.19
	SSP5-8.5	3604.02	3030.73	435.37	299.52	3.67	11.76	8.09
2041–2060	SSP1-2.6	3669.12	3085.48	381.07	272.32	2.94	10.29	7.35
	SSP3-7.0	3667.86	3007.47	461.03	273.81	5.06	12.45	7.39
	SSP5-8.5	3729.25	3142.96	326.15	355.08	−0.78	8.8	9.59
2061–2080	SSP1-2.6	3739.33	3009.64	367.67	335.66	0.86	9.93	9.06
	SSP3-7.0	3545.25	3031.41	438.34	324.85	3.06	11.84	8.77
	SSP5-8.5	3796.69	3091.39	379.9	381.84	0.05	10.26	10.31
2081–2100	SSP1-2.6	3822.05	3159.47	308.38	372.8	−1.74	8.33	10.07
	SSP3-7.0	3880.64	3060.13	409.66	547.72	−3.73	11.06	14.79
	SSP5-8.5	3814.84	3001.51	467.77	414.25	1.45	12.63	11.19

*: Net change (%) = (Contraction–Expansion)/Current area × 100%. Positive values in the “Net change (%)” column indicate net range contraction; negative values indicate net range expansion.

## Data Availability

The original data used for analysis in this study are all publicly available from online sources. Climate data were obtained from the WorldClim database (https://worldclim.org accessed on 25 October 2025). Soil data were derived from the Harmonized World Soil Database (HWSD) grid dataset provided by the Food and Agriculture Organization of the United Nations (http://www.fao.org/soils-portal accessed on 25 October 2025).

## Supplementary Materials

The following supporting information can be downloaded at: https://www.mdpi.com/article/10.3390/biology15120916/s1, Figure S1: Workflow diagram of the study methodology; Figure S2: Pearson correlation analysis; Table S1: MaxEnt model performance (AUC) under current and future climate scenarios across periods.
