# Supplementary figures and images for: Prediction of the Global Potential Distribution Area of Phytopythium litorale Based on the Maxent Model
Source: Biology (Basel). 2026 Jun 11;15(12):916. doi: 10.3390/biology15120916 (PMC13295806; doi:10.3390/biology15120916)

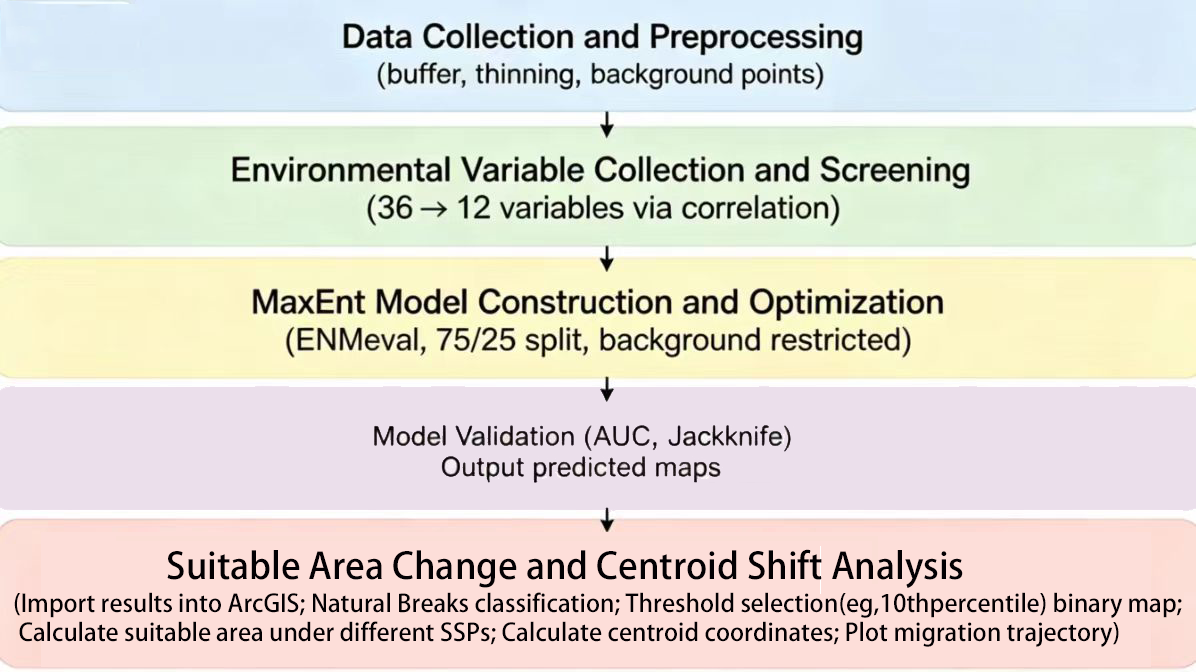

Supplement: Supplementary file 1 [file biology-15-00916-s001.zip › Figure S1.tif]

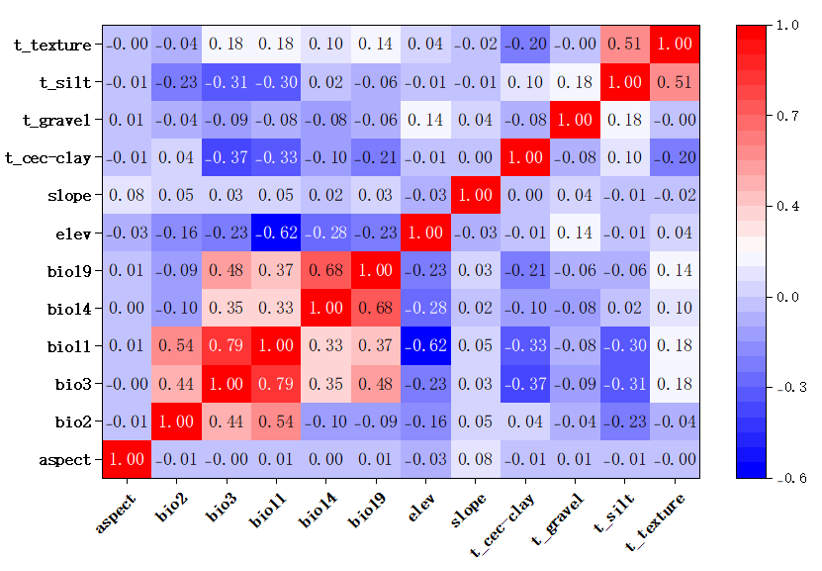

Supplement: Supplementary file 1 [file biology-15-00916-s001.zip › Figure S2.tif]
